# Novel Polymorphisms and Genetic Characteristics of the Prion Protein Gene (*PRNP*) in Dogs—A Resistant Animal of Prion Disease

**DOI:** 10.3390/ijms21114160

**Published:** 2020-06-10

**Authors:** Dong-Ju Kim, Yong-Chan Kim, An-Dang Kim, Byung-Hoon Jeong

**Affiliations:** 1 Korea Zoonosis Research Institute, Jeonbuk National University, Iksan, Jeonbuk 54531, Korea; wizardnight@jbnu.ac.kr (D.-J.K.); kych@jbnu.ac.kr (Y.-C.K.); 2 Department of Bioactive Material Sciences and Institute for Molecular Biology and Genetics, Jeonbuk National University, Jeonju, Jeonbuk 54896, Korea; 3 Cool-Pet Animal Hospital, Anyang, Gyeonggi 14066, Korea; kad7582@hanmail.net

**Keywords:** dog, prion, PRNP, octapeptide, polymorphism, SNP

## Abstract

Transmissible spongiform encephalopathies (TSEs) have been reported in a wide range of species. However, TSE infection in natural cases has never been reported in dogs. Previous studies have reported that polymorphisms of the prion protein gene (*PRNP*) have a direct impact on the susceptibility of TSE. However, studies on polymorphisms of the canine *PRNP* gene are very rare in dogs. We examined the genotype, allele, and haplotype frequencies of canine *PRNP* in 204 dogs using direct sequencing and analyzed linkage disequilibrium (LD) using Haploview version 4.2. In addition, to evaluate the impact of nonsynonymous polymorphisms on the function of prion protein (PrP), we carried out *in silico* analysis using PolyPhen-2, PROVEAN, and PANTHER. Furthermore, we analyzed the structure of PrP and hydrogen bonds according to alleles of nonsynonymous single nucleotide polymorphisms (SNPs) using the Swiss-Pdb Viewer program. Finally, we predicted the impact of the polymorphisms on the aggregation propensity of dog PrP using AMYCO. We identified a total of eight polymorphisms, including five novel SNPs and one insertion/deletion polymorphism, and found strong LDs and six major haplotypes among eight polymorphisms. In addition, we identified significantly different distribution of haplotypes among eight dog breeds, however, the kinds of identified polymorphisms were different among each dog breed. We predicted that *p*.64_71del HGGGWGQP, Asp182Gly, and Asp182Glu polymorphisms can impact the function and/or structure of dog PrP. Furthermore, the number of hydrogen bonds of dog PrP with the Glu182 and Gly182 alleles were predicted to be less than those with the Asp182 allele. Finally, Asp163Glu and Asp182Gly showed more aggregation propensity than wild-type dog PrP. These results suggest that nonsynonymous SNPs, Asp182Glu and Asp182Gly, can influence the stability of dog PrP and confer the possibility of TSE infection in dogs.

## 1. Introduction

Transmissible spongiform encephalopathies (TSEs), also known as prion diseases, are neurodegenerative diseases caused by conversion of the normal prion protein (PrP^C^) into aggregated, self-propagating and disease-associated isoforms (PrP^Sc^). TSE has been reported in a wide range of species, such as goats, sheep, cattle, mink, cats, and humans [1,2,3,4,5,6,7,8,9,10,11]. However, during the outbreak of bovine spongiform encephalopathy (BSE) in the UK, BSE was transmitted to cats through contaminated food. Although dogs were equally likely to have been exposed to BSE-contaminated food, TSE infection was never reported in dogs [12,13]. Several studies have been performed to explain the mechanism of resistance to TSEs in dogs. Dog-specific amino acids (Asp163) of dog PrP were observed using multiple sequence alignment of PrP among several species. *In silico* studies have predicted that Asp163 can contribute to the stability of dog PrP by expansion of helix 1 and negative surface charge [14,15]. A circular dichroism study reported that the PrP of dogs is more stable than that of prion disease-susceptible animals, including hamsters and mice, at four pH values [16]. Madin–Darby canine kidney (MDCK) cells, which are derived from dogs, have been shown to be resistant to TSE infection [17,18]. In a study using protein misfolding cyclic amplification (PMCA), dog brain homogenate showed resistance to several prion agents, including BSE, scrapie, and chronic wasting disease (CWD) [19]. The dog-specific amino acid (Asp163), which is located on the α1-β2 loop of PrP-transgenic mice, showed complete resistance to TSEs following intracerebral inoculation with three mouse-adapted scrapie strains, including Rocky Mountain Laboratory (RML), 301C, and 22L [18].

In previous studies, genetic polymorphisms of the prion protein gene (*PRNP*) were associated with the susceptibility of several types of prion diseases. In sheep, scrapie risk-groups have been defined according to the composition of haplotypes at codons 136, 154, and 171 of ovine *PRNP* [2,20,21]. In goats, codons Ile142Met, His143Arg, Asn146Ser, Arg211Gln, and Gln222Lys of the caprine *PRNP* gene were linked to the protection against scrapie development [22,23,24,25]. In humans, the *PRNP* codon 129 single nucleotide polymorphism (SNP) is strongly associated with variant and sporadic Creutzfeldt–Jakob disease (CJD) [26]. In cattle, 23 bp and 12 bp indel polymorphisms at the promotor region and intron 1 were related to the susceptibility of BSE, respectively [27,28,29]. Therefore, it is very important to investigate polymorphisms associated with the susceptibility and/or resistance of TSEs because amino acid substitutions of proteins can influence the structure and/or function of PrP [30]. A previous study reported that the stabilization of helix 2 in the recombinant mouse prion protein leads to an increase in the stability of the protein. Stabilization of the local region of prion protein slowed down the formation of oligomers and amyloid fibrils [31]. Therefore, SNPs that can modulate the stability of prion proteins have been reported to be related to genetic susceptibility in several kinds of TSEs. In dogs, three nonsynonymous SNPs, including Gly101Ser, Asp163Glu, and Thr169Ile, have been reported in a small sample size of mixed dogs [32]. Of the three nonsynonymous SNPs, only the Asp163Glu SNP has been linked with resistance to TSE.

In the present study, we investigated the genotype, allele and haplotype frequencies of the *PRNP* gene in 204 dogs, which were composed of eight dog breeds, including Maltese, Shih Tzu, Toy Poodle, Yorkshire Terrier, Pomeranian, Chihuahua, Schnauzer, Bichon Frise, and mixed dogs. In addition, we compared tandem repeat domains among several species and the distribution of haplotypes of canine *PRNP* gene polymorphisms among eight breeds. Furthermore, we evaluated the impact of polymorphisms of the canine *PRNP* gene on dog PrP using PolyPhen-2, PROVEAN, PANTHER, and AMYCO [33,34,35,36,37]. Finally, we predicted the structure of prion proteins and hydrogen bonds according to alleles of nonsynonymous SNPs using the Swiss-Pdb Viewer program.

## 2. Results

### 2.1. Identification of Polymorphisms in Canine PRNP and Genetic Analysis

**Figure 1:**
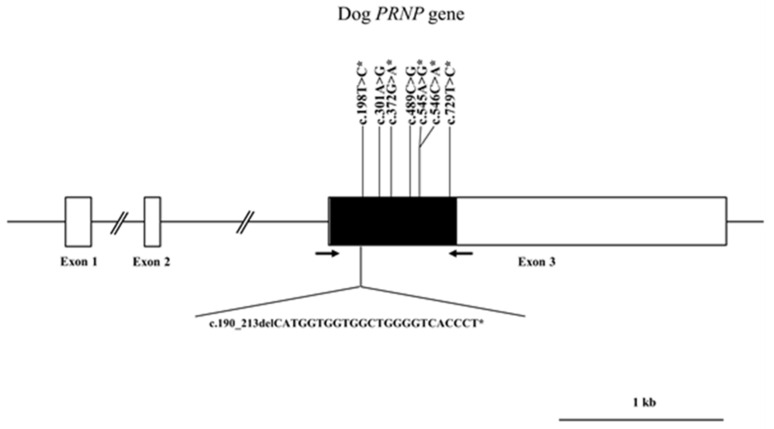
Gene map of polymorphisms identified in the canine prion protein (*PRNP*) gene on chromosome 24. The open reading frame (ORF) is indicated by a shaded block, and the 5′ and 3′ untranslated regions (UTRs) are indicated by white blocks. Arrows indicate the regions sequenced. The Y-shaped bar indicates the octapeptide deletion polymorphisms identified in the canine *PRNP* gene. Asterisks indicate the novel polymorphisms found in this study.

**Figure 2:**
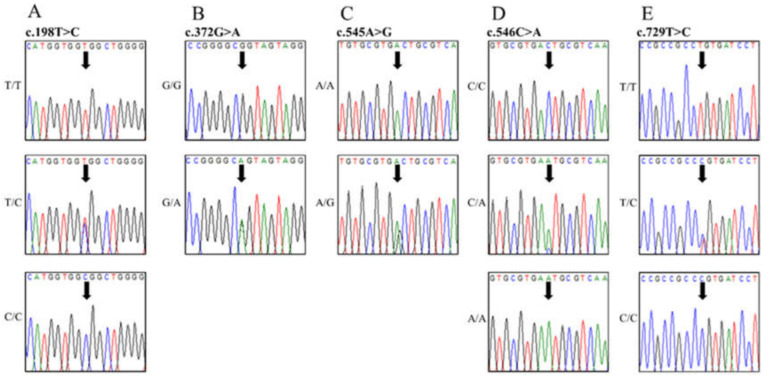
Electropherograms of 5 novel single nucleotide polymorphisms (SNPs) of the canine prion protein (*PRNP*) gene found in dogs. (**A**) Electropherograms showing the three genotypes at polymorphic codon 66 (Gly66Gly). Upper portion GGT/GGT, middle portion: GGT/GGC, and lower portion: GGC/GGC. (**B**) Electropherograms showing the two genotypes at polymorphic codon 124 (Ala124Ala). Upper portion: GCG/GCG and lower portion: GCG/GCA. (**C**) Electropherograms showing the two genotypes at polymorphic codon 182 (Asp182Glu). Upper portion: GAC/GAC and lower portion: GAC/GGC. (**D**) Electropherograms showing the three genotypes at polymorphic codon 182 (Asp182Gly). Upper portion: GAC/GAC, middle portion: GAC/GAA, and lower portion GAA/GAA. (**E**) Electropherograms showing the three genotypes at polymorphic codon 243 (Pro243Pro). Upper portion: CCT/CCT, middle portion: CCT/CCC, and lower portion: CCC/CCC.

**Figure 3:**
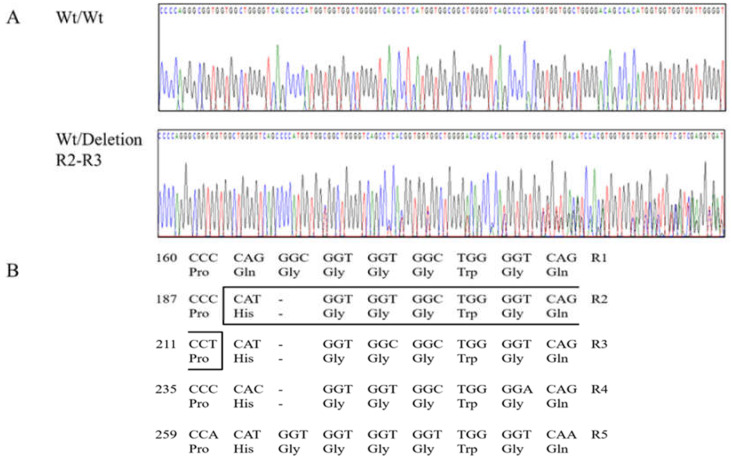
Electropherograms of novel insertion/deletion polymorphism of the canine prion protein (*PRNP*) gene. (**A**) Electropherograms showing the polymorphism at the octapeptide repeat region. Upper portion: insertion/insertion homozygote of the canine *PRNP* and lower portion: insertion/deletion heterozygote of the canine *PRNP*. (**B**) The amino acid and nucleotide sequences of the repeat region of the canine *PRNP* gene. The box indicates the 64_71del HGGGWGQP polymorphism which was found between R2 and R3. R1: octapeptide repeat 1 (54Pro, 55Gln, 56Gly, 57Gly, 58Gly, 59Gly, 60Trp, 61Gly, 62Gln); R2: octapeptide repeat 2 (63Pro, 64His, 65Gly, 66Gly, 67Gly, 68Trp, 69Gly, 70Gln); R3: octapeptide repeat 3 (71Pro, 72His, 73Gly, 74Gly, 75Gly, 76Trp, 77Gly, 78Gln); R4: octapeptide repeat 4 (79Pro, 80His, 81Gly, 82Gly, 83Gly, 84Trp, 85Gly, 86Gln); and R5: octapeptide repeat 5 (87Pro, 88His, 89Gly, 90Gly, 91Gly, 92Gly, 93Trp, 94Gly, 95Gln).

The canine *PRNP* gene is composed of three exons. To investigate the genotype and allele frequencies of the canine *PRNP* gene polymorphisms, we performed a Polymerase chain reaction (PCR) to amplify the open reading frame (ORF) region of the canine *PRNP* gene in exon 3. A total of 204 dogs were analyzed, and all sequences of amplicons (959 bp) were identical to the *PRNP* gene of *Canis lupus familiaris* registered in GenBank (AF042843). We identified seven SNPs and one insertion/deletion in the ORF region (Figure 1). Except for previously reported 301A>G (Ser101Gly) and c.489C>G (Asp163Glu), we identified six novel polymorphisms, including one insertion/deletion (c.190_213delCATGGTGGTGGCTGGGGTCACCCT, 64_71del HGGGWGQP), two nonsynonymous SNPs (c.545A>G, Asp182Gly; c.546C>A, Asp182Glu), and three synonymous SNPs (c.198T>C, Gly66Gly; c.372G>A, Ala124Ala; c.729T>C, Pro243Pro) (Figure 2 and Figure 3). The genotype and allele frequencies of polymorphisms of the canine *PRNP* gene are described in Table 1. Except for 198T>C and c.546C>A, the genotype frequencies of all six polymorphisms were in Hardy–Weinberg equilibrium (HWE). The insertion/deletion was located in the octapeptide region between R2 and R3 (Figure 3). We performed haplotype analysis of 8 polymorphisms of the canine *PRNP* gene (Table 2). The WtTAGCACT haplotype was observed with the highest frequency (0.696) in the canine *PRNP* gene. We also investigated the linkage disequilibrium (LD) values among the eight canine *PRNP* polymorphisms with (|D’|) and r^2^ values (Table 3). Strong LD was observed (*r^2^* > 0.333) for c.198T>C with c.301A>G, c.301A>G with c.489C>G, c.301A>G with c.729T>C and c.489C>G with c.729T>C. These LD groups were also in strong LD with D’ values of 0.9–1.0.

### 2.2. Comparison of Tandem Repeat Domains of PrP Among Several Species

**Figure 4:**
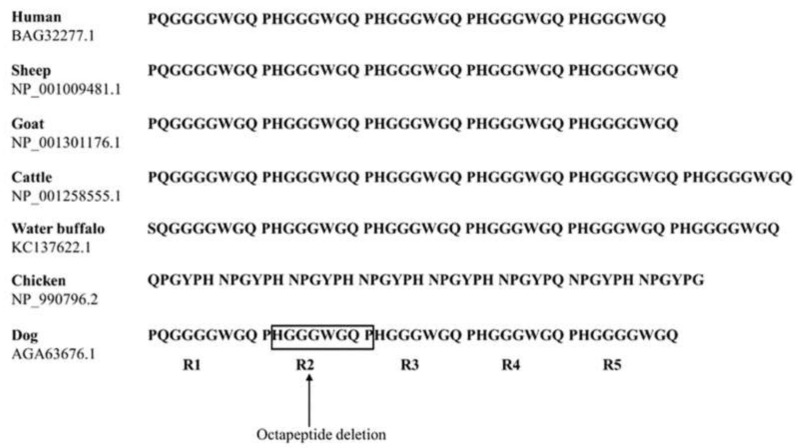
Comparison of tandem repeat sequences in humans, sheep, water buffalos, goats, cattle, chickens, and dogs. Octapeptide repeat sequences were obtained from GenBank at the National Center for Biotechnology Information (NCBI), including human (*Homo sapiens*, BAG32277.1), sheep (*Ovis aries*, NP_001009481.1), goat (*Capra hircus*, NP_001301176.1), cattle (*Bos taurus*, NP_001258555.1), water buffalo (*Bubalus bubalis*, KC137622.1), chicken (*Gallus gallus*, NP_990796.2), and dog (*Canis lupus familiaris*, AGA63676.1). The arrow indicates the octapeptide repeat deletion polymorphism found in this study. R1 ~ R5 indicate octapeptide repeat regions of dog PrP. R1: octapeptide repeat 1 (54Pro, 55Gln, 56Gly, 57Gly, 58Gly, 59Gly, 60Trp, 61Gly, 62Gln); R2: octapeptide repeat 2 (63Pro, 64His, 65Gly, 66Gly, 67Gly, 68Trp, 69Gly, 70Gln); R3: octapeptide repeat 3 (71Pro, 72His, 73Gly, 74Gly, 75Gly, 76Trp, 77Gly, 78Gln); R4: octapeptide repeat 4 (79Pro, 80His, 81Gly, 82Gly, 83Gly, 84Trp, 85Gly, 86Gln); and R5: octapeptide repeat 5 (87Pro, 88His, 89Gly, 90Gly, 91Gly, 92Gly, 93Trp, 94Gly, 95Gln).

Previous studies have been reported that insertion/deletion located on tandem repeat region of PrP is related to the progression of prion diseases [38,39]. In addition, since tandem repeat region has been associated with metal ion binding ability, the variability of amino acid composition and the length of tandem repeats in several species showed the diversity of physiological function of PrP [40,41,42,43]. Thus, to find distinct feature of tandem repeat domain of dog PrP, we compared the amino acid sequence of tandem repeats among various animals including humans, sheep, goats, cattle, water buffalos, chickens, and dogs (Figure 4).

The octapeptide region of dogs is identical to that of goats and sheep. The octapeptide region of cattle was composed of six repeats containing an additional octapeptide repeat unit, R5 (PHGGGWGQ), compared to that of dog PrP [44]. The human octapeptide repeat, R5 (PHGGGWGQ) is composed of one less glycine than dog PrP (PHGGGGWGQ). The octapeptide repeat of water buffalos showing unique R1 (SQGGGGWFQ) and R5 (PHGGGWGQ) is composed of one less glycine compared to that of dog PrP (PHGGGGWGQ). The tandem repeat (QPGYPH) of chickens is composed of a hexapeptide repeat, which is significantly different from that of dog PrP. The hexapeptide repeat of chickens also has a different length of tandem repeats compared to dog PrP (chicken: 48 aa; dog: 42 aa).

### 2.3. Comparison of the Distribution of the Haplotypes of PRNP Polymorphisms in Eight Dog Breeds

**Figure 5:**
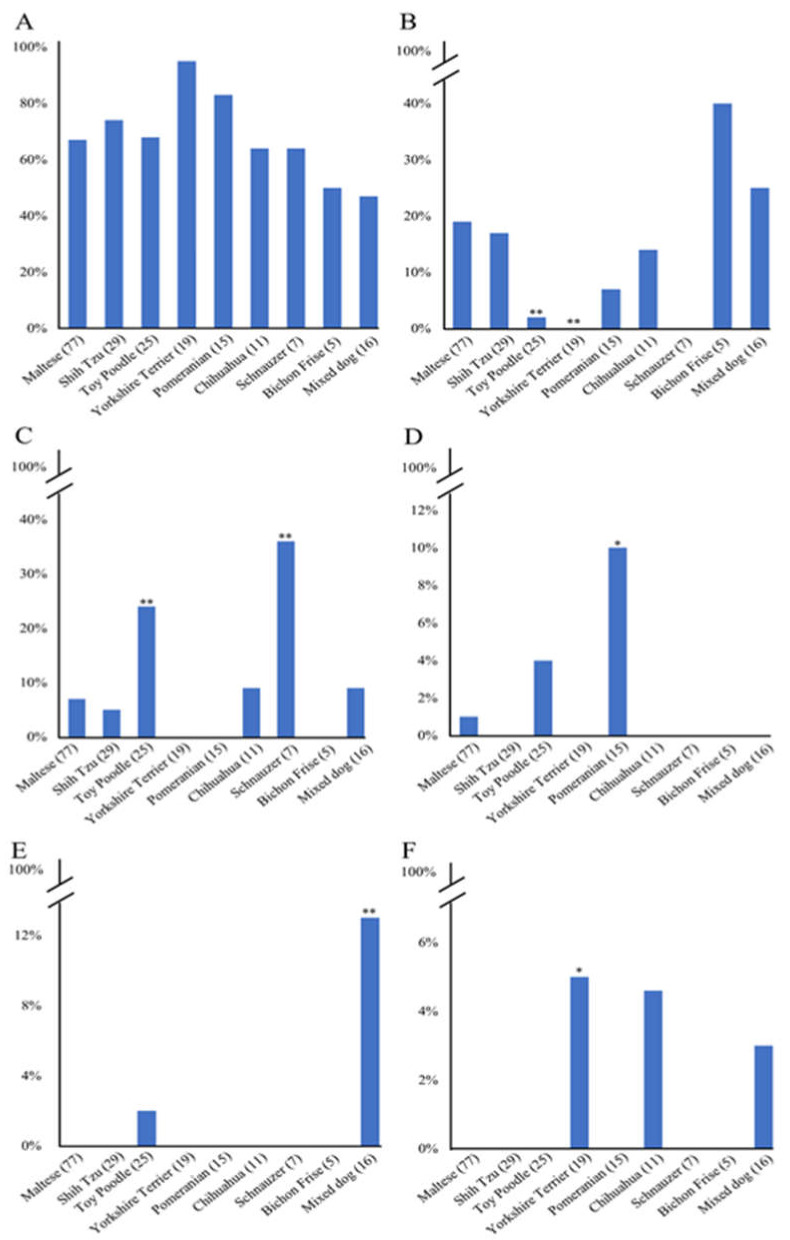
Comparison of the distribution of haplotypes according to dog breeds. (**A**) Comparison of the distribution of haplotype 1 among 8 dog breeds. (**B**) Comparison of the distribution of haplotype 2 between Maltese and other breeds. (**C**) Comparison of the distribution of haplotype 3 between Maltese and other breeds. (**D**) Comparison of the distribution of haplotype 4 between Maltese and other breeds. (**E**) Comparison of the distribution of haplotype 5 between Maltese and other breeds. (**F**) Comparison of the distribution of haplotype 6 between Maltese and other breeds. Parentheses indicate each number of dog breeds. * indicates *p* < 0.05. ** indicates *p* < 0.01.

We compared the distribution of haplotypes of the canine *PRNP* gene among eight dog breeds (Figure 5). In brief, the distribution of haplotype 1 was not significantly different among the eight dog breeds (Figure 5A). However, the distribution or haplotypes 2, 3, 4, 5, and 6 were significantly different among the eight dog breeds. In detail, haplotype 2 frequency of Maltese was significantly different from that of Toy poodle (*p* < 0.01, Figure 5B) and Yorkshire Terrier (*p* < 0.01). Haplotype 3 frequency of Maltese was significantly different from that of Toy poodle (*p* < 0.01) and Schnauzer (*p* < 0.01, Figure 5C). Haplotype 4 frequency of Maltese was significantly different from that of Pomeranian (*p* < 0.05, Figure 5D). Haplotype 5 frequency of Maltese was significantly different from that of Mixed dogs (*p* < 0.01, Figure 5E). Haplotype 6 frequency of Maltese was significantly different from that of Yorkshire Terrier (*p* < 0.05, Figure 5F).

### 2.4. The Number of Canine PRNP Polymorphisms in Eight Breeds

We investigated the number of polymorphisms found in dog breeds (Table 4). In brief, 8 polymorphisms of the *PRNP* gene found in 77 Maltese dogs were 64_71delHGGGWGQP, Gly66Gly, Ser101Gly, Ala124Ala, Asp163Glu, Asp182Gly, Asp182Glu and Pro243Pro. Five polymorphisms of the *PRNP* gene found in 29 Shih Tzu dogs were Gly66Gly, Ser101Gly, Ala124Ala, Asp163Glu, and Pro243Pro. Six polymorphisms of the *PRNP* gene found in 25 Toy Poodle dogs were 64_71delHGGGWGQP, Gly66Gly, Ser101Gly, Ala 124Ala, Asp163Glu, and Pro243Pro. One polymorphism of the *PRNP* gene found in 19 Yorkshire Terrier dogs was Asp163Glu. Four polymorphisms of the *PRNP* gene found in 15 Pomeranian dogs were Gly66Gly, Ser101Gly, Asp163Glu, and Pro243Pro. Six polymorphisms of the *PRNP* gene found in 11 Chihuahua dogs were Gly66Gly, Ser101Gly, Asp163Glu, Asp182Gly, Asp182Glu, and Pro243Pro. Four polymorphisms of the *PRNP* gene found in four Schnauzer dogs were Gly66Gly, Ser101Gly, Asp163Glu, and Pro243Pro. Three polymorphisms of the *PRNP* gene found in three Bichon Frise dogs were Ser101Gly, Asp163Glu, and Pro243Pro. Six polymorphisms of the *PRNP* gene found in mixed dogs were Gly66Gly, Ser101Gly, Ala 124Ala, Asp163Glu, Asp182Glu, and Pro243Pro. Collectively, the number of polymorphisms showed the diversity from 1 to 8. In brief, the dog breed with the lowest number of polymorphisms was the Yorkshire Terrier (1). In addition, the dog breed with the highest number of polymorphisms was the Maltese (8), followed by Toy poodle (6), Chihuahua (6), and Mixed dog (6)

### 2.5. Estimation of the Functional Effect of Genetic Polymorphisms of Dog PrP

We estimated the impact of nonsynonymous SNPs and insertion/deletion of the *PRNP* gene on dog PrP using PolyPhen-2, PROVEAN, and PANTHER. Detailed scores predicted by the three programs are described in Table 5. In brief, an octapeptide deletion (64_71del HGGGWGQP) was estimated to be “Deleterious” by PROVEAN. Histidine residue of octapeptide interacts with copper ion and plays a pivotal role in function of octapeptide repeat region. The octapeptide repeat region is related to protection against oxidative stress, N-methyl-D-aspartate receptor activity, glutamate uptake, and copper homeostasis mediated by the binding ability of metal ion [41,45]. Thus, the deletion allele of insertion/deletion can be deleterious to normal physiological function of dog PrP. Ser101Gly and Asp163Glu SNPs were predicted to be ‘Benign’, ‘Neutral’, and ‘Probably benign’ by PolyPhen-2, PROVEAN, and PANTHER, respectively. Interestingly, Asp182Gly and Asp182Glu were predicted to be ‘Probably damaging’ by PolyPhen-2 and PANTHER. However, Asp182Gly and Asp182Glu were predicted to be ‘Neutral’ by PROVEAN (Table 5).

### 2.6. Prediction of the Structural Alteration of Dog PrP Induced by Nonsynonymous SNPs

**Figure 6:**
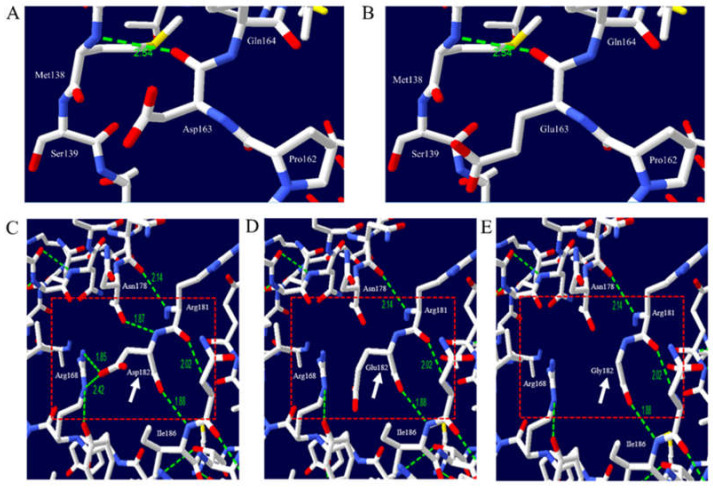
Prediction of 3D structure and hydrogen bonds of dog prion protein (PrP). The white arrow indicates target amino acid residues. The red box indicates adjacent amino acid residues. The green dotted line indicates hydrogen bonds. The green numbers indicate the distance of the hydrogen bonds. (**A**) 3D structure of dog PrP with allele Asp163, (**B**) 3D structure of dog PrP with allele Glu163, (**C**) 3D structure of dog PrP with allele Asp182, (**D**) 3D structure of dog PrP with allele Glu182, and (**E**) 3D structure of dog PrP with allele Gly182.

The 3D structure of dog PrP was visualized according to alleles of the nonsynonymous SNPs of the canine *PRNP* gene (Figure 6). We analyzed the hydrogen bonds of dog PrP with the Asp163 and Glu163 alleles. Asp163 was predicted to have one hydrogen bond (2.54 Å) with Met138 (Figure 6A). Glu163 was also predicted to have an identical length of hydrogen bond (2.54 Å) with Met138 (Figure 6B). We identified two nonsynonymous SNPs (Asp182Gly and Asp182Glu) located on helix 2. Asp182 was predicted to have two hydrogen bonds (1.85 and 2.42 Å) with Arg168 and two hydrogen bonds with Ile186 (1.88 Å) and Asn178 (1.87 Å) (Figure 6C). Glu182 was predicted to have one hydrogen bond with Ile186 (1.88 Å) (Figure 6D). Gly182 was also predicted to have one hydrogen bond with Ile186 (1.88 Å) (Figure 6E).

### 2.7. Evaluation of Polymorphisms on the Aggregation Propensity of Dog PrP

**Figure 7:**
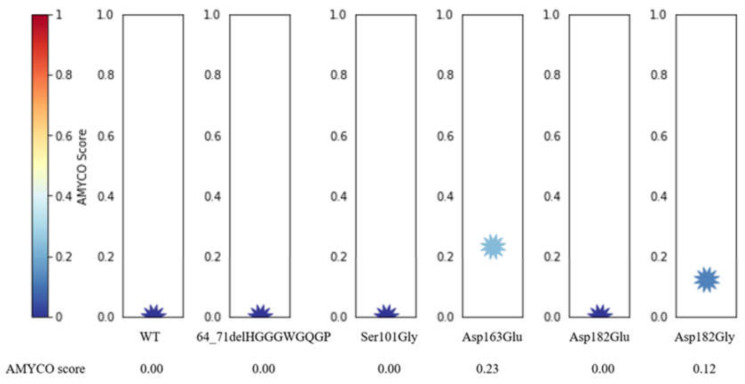
Prediction of aggregation propensity according to alleles of dog prion protein (PrP) polymorphisms. The impact of polymorphisms on the aggregation propensity of dog PrP was evaluated as values from 0.0 to 1.0 by AMYCO analysis. AMYCO scores <0.45 and >0.78 indicate low and high aggregation propensities of the protein, respectively.

To estimate the impact of the polymorphism of the canine *PRNP* gene on the aggregation propensity of dog PrP, we utilized the AMYCO program. Dog PrPs with 64_71delHGGGWGQGP, Ser101Gly, and Asp182Glu were predicted to score 0. Dog PrP with Glu163 (score 0.23) showed more aggregation propensity than that with Asp163. Dog PrP with Gly182 (score 0.12) showed more aggregation propensity than that with Asp182 (Figure 7).

## 3. Discussion

Natural interspecies transmission of TSEs has never been reported in rabbits, horses, pigs, and dogs. However, the number of TSE-resistant species has decreased with the reporting of prion diseases by experimental infections. In pigs, experimental transmission of BSE via intracerebral (IC) injection and intraperitoneal (IP) injection was reported [46,47,48], and pig PrP transgenic mice showed BSE infection via IC injection [49]. In addition, experimental transmission of RML, ME7, 22L, 139A, 79A, 22F, CWD, BSE, SSBP/1, and CH1641 using PMCA was also reported in rabbit. Furthermore, rabbit PrP transgenic mice were infected by BSE, BSE-L, *de novo* New Zealand White (NZW), ME7 and RML. However, dog PrP showed resistance against, several prion agents including BSE, scrape, CWD, ME7, RML, 22F, 22L 87V, 22A, 79A, and 139A, in seeded PMCA [50]. MDCKcells showed resistance to the RML strain, and dog-specific amino acid N158D transgenic mice also showed resistance to infection of three different mouse prion strains, including RML, 301C, and 22L [17,18]. These results indicate that dogs are prion disease-resistant animals. However, these studies did not consider genetic polymorphisms of dog PrP.

Since polymorphism of the *PRNP* gene has been associated with the susceptibility to prion diseases [5,11,51,52], we amplified the ORF region of the canine *PRNP* gene to identify the genetic polymorphism of this gene. We identified a total of eight polymorphisms, including two novel nonsynonymous SNPs and one insertion/deletion (Figure 1). We identified strong LDs and six major haplotypes among eight polymorphisms. The distribution of haplotypes was significantly different among the eight dog breeds. In addition, the number of identified polymorphisms was different from each dog breed (Table 4). Notably, Yorkshire Terrier showed the lowest number of polymorphisms in dog breeds with more than 12 samples capable of excavating 1% frequencies of SNPs with 96% probability (Table 4). Since the wolf and dog PrPs have the same amino acid sequence, the evolutionary distance of the *PRNP* gene between dog and wolf can be estimated according to the number of polymorphisms. In comparison with Maltese, Shih Tzu, Toy Poodle, and Pomeranian, which showed highly polymorphic *PRNP* gene, Yorkshire Terrier is presumed to be a close evolutionary distance of the *PRNP* gene with wolf.

We also estimated the impact of polymorphisms on dog PrP using PolyPhen-2, PROVEAN, and PANTHER. All three *in silico* programs predicted that Asp163Glu was benign. A previous study reported that Asp163Glu did not influence the susceptibility to TSE of transgenic mice expressing dog-specific amino acids 158Asp and 158Glu [53]. Codon 158 in mouse PrP is equivalent to codon 163 in dog PrP. In the present study, we observed similar results using *in silico* programs in which Asp163Glu does not impact the structure and/or function of dog PrP. Notably, PROVEAN and PANTHER predicted that the *p*.64_71del HGGGWGQP, Asp182Gly, and Asp182Glu polymorphisms can impact the function and/or structure of dog PrP (Table 5). These estimations suggested the possibility that *p*.64_71del HGGGWGQP, Asp182Gly, and Asp182Glu can impact the susceptibility of dogs to TSE (Table 5). However, Asp182Gly and Asp182Glu were predicted as neutral using PROVEAN. Because PROVEAN was estimated using clustering of basic local alignment search tool (BLAST) and comparing homologs collected from a database, PROVEAN predicted that Asp182Gly and Asp182Glu did not impact the function of PrP.

Next, we predicted the 3D structure of dog PrP to evaluate the impact of three nonsynonymous SNPs, including Asp163Glu, Asp182Glu, and Asp182Gly. We compared the distribution of hydrogen bonds between alleles Asp163 and Glu163 of dog PrP. The distribution of hydrogen bonds in dog PrP is identical between the Asp163 and Glu163 alleles (Figure 6A,B). Dog PrP with Asp182 was predicted to have four hydrogen bonds. However, dog PrP with Glu182 and Gly182 was predicted to have only one hydrogen bond (Figure 6C,D). The number of hydrogen bonds can affect the stability and structure of proteins [54,55,56]. Because the stability of PrP is related to the susceptibility of prion disease, Asp182Glu and Asp182Gly SNPs of the canine *PRNP* gene can influence the susceptibility to TSE of dogs. We estimated the impact of the polymorphism of the canine *PRNP* gene on the aggregation propensity of dog PrP and found that dog PrP with Asp163Gly and Asp182Gly (score 0.12) had a higher aggregation propensity than that of wild-type dog PrP (Figure 7). Collectively, Asp182Glu, and Asp182Gly are presumed to be deleterious. Based on our analysis, Shih Tzu, Toy Poodle, and Pomeranian, which do not carry Asp182Glu and Asp182Gly, are presumed to be resistant to prion disease compared to Maltese and Yorkshire Terrier in dog breeds with more than 12 samples. It indicates that evolutionary sensitization to prion infection can be occurred in Maltese and Yorkshire Terrier. To confirm the impact of Asp182Glu and Asp182Gly SNPs on the susceptibility to prion disease of dogs, infection experiments with prion agents will be necessary in MDCK cells and transgenic mice expressing dog PrP with two amino acid substitutions, Asp182Glu and Asp182Gly, in the future.

Although most of our analysis has been focused on nonsynonymous SNPs, there are recent evidences that synonymous SNPs introduce less commonly used codons, which may alter the speed of translation and ultimately folding, function, and stability of the mature protein [57,58]. Since prion diseases are induced by misfolded prion protein, these are important considerations of synonymous SNPs. Further study of synonymous SNP is highly desirable in the future.

## 4. Materials and Methods

### 4.1. Ethics Statement

Whole blood samples from 204 dogs were provided by the Anyang cool pet animal hospital in the Republic of Korea. The samples were collected depending upon hospital visit rates based on the prevalence of dog breed in the Republic of Korea. The total of 204 dogs consisted of 8 dog breeds, including 77 Maltese (37.75%), 29 Shih Tzu (14.22%), 25 Toy Poodle (12.25%), 19 Yorkshire Terrier (9.31%), 15 Pomeranian (7.35%), 11 Chihuahua (5.39%), 7 Schnauzer (3.43%), 5 Bichon Frise (2.45%), and 16 mixed dog (7.84). Mixed dogs are cross-breed dogs obtained by breeding between a Maltese and a Toy poodle. The origin of mixed dogs was determined by pedigree document. All experimental procedures were accredited by the Institute of Animal Care and Use Committee of Jeonbuk National University (JBNU 2018-062).

### 4.2. Genomic DNA Extraction and Genetic Analysis

Genomic DNA was isolated from whole blood samples of 204 dogs using a Hi Yield Genomic DNA Mini Kit (Real Biotech Corporation, Taipei, Taiwan) and a Bead Genomic DNA Prep Kit (Biofact, Daejeon, Korea) following the manufacturer’s instructions. PCR was carried out using gene-specific sense and antisense primers: canine *PRNP*-F (TGTGCAGATGTTCTCGCTGT) and canine *PRNP*-R (GAAGCGGGAATGAGACACCA). These primers were designed to amplify the entire ORF region of the canine *PRNP* gene. We performed PCR using BioFACT™ *Taq* DNA Polymerase (Biofact, Daejeon, Korea). The 25 µL PCR mixture was composed of 5 µL of 10× DNA polymerase buffer, 2.5 units of 10× *Taq* DNA polymerase, 1 µL of genomic DNA, 10 pmol of each primer, and 0.5 µL of a 0.2 M dNTP mixture. The PCR conditions were as follows: denaturing at 95 °C for 2 min, followed by 34 cycles of 95 °C for 20 s, 62 °C for 30 s, and 72 °C for 1 min, and 30 s and 1 cycle of 72 °C for 5 min. All PCR products were analyzed by electrophoresis on a 1.0% agarose gel stained with ethidium bromide (EtBr). PCR products were directly sequenced using an ABI 3730 sequencer (ABI, Foster City, California, USA). Sequencing results were read using Finch TV software (Geospiza Inc, Seattle, WA, USA).

### 4.3. Statistical Analysis

The chi-squared test was used to determine whether polymorphisms of the canine *PRNP* gene were in HWE and to determine whether there were differences in difference of allele frequencies among the 8 dog breeds. We examined LD and analyzed haplotypes of polymorphisms of the canine *PRNP* gene. Lewontin’s D′ (|D′|), coefficient *r*^2^ values and haplotypes of polymorphisms of the canine *PRNP* gene were analyzed using Haploview version 4.2 (Broad Institute, Cambridge, MA, USA).

### 4.4. Prediction of Protein Functional Alterations in Dog PrP

PolyPhen-2 (http://genetics.bwh.harvard.edu/pph2/index.shtml), PROVEAN (http://provean.jcvi.org/seq_submit.php) and PANTHER (http://www.pantherdb.org) predicted the impact on protein function induced by variations in protein sequence in dog PrP. The prediction of PolyPhen-2 was based on the number of features containing the sequence, structural, and phylogenetic information characterizing the variation. PolyPhen-2 was predicted to be benign, possibly damaging, or probably damaging based on pairs of false positive rate (FPR) thresholds with scores ranging from 0.0 to 1.0. PROVEAN estimates the impact according to protein sequence variations on protein function. The PROVEAN scores are computed by clustering BLAST hits according to the homologs collected from a database (the NCBI nr database). The top 30 clusters of closely related sequences form the supporting sequence set, which will be used to make the prediction. PROVEAN scores below –2.5 indicate “deleterious,” and above –2.5 indicates “neutral”. PANTHER was based on evolutionary preservation. Homologous proteins are used to reconstruct the likely sequences of ancestral proteins at nodes in a phylogenetic tree, and each amino acid can be tracked back in time from its present state to estimate how long that state has been conserved in its ancestors [38]. PANTHER calculates preservation time to evaluate the substitution of amino acids. The interpretation of preservation time is described as follows: “Probably damaging” is greater than 450my; “Possibly damaging” is between 200my and 450my; “Probably benign” is less than 200my. AMYCO (http://bioinf.uab.cat/amyco/) can evaluate the impact of polymorphism on the aggregation propensity of proteins. AMYCO calculated the impact of amyloidogenic sequences and the contribution of composition to the aggregation of dog PrP using the pWALTZ and PAPA algorithms. This is the interpretation of the PSEP score.

### 4.5. 3D Structure Modeling of Dog PrP

The 3D structure model of dog PrP using nuclear magnetic resonance (NMR) spectroscopy was obtained from the Protein Data Bank (http://www.rcsb.org/structure/1XYK) (Protein ID: 1XYK). Analysis of the impact of the nonsynonymous SNPs on dog prion protein was performed by the Swiss-PdbViewer program (https://spdbv.vital-it.ch/). The models of nonsynonymous SNPs of the canine *PRNP* gene were generated at Asp163Glu, Asp182Gly, and Asp182G. Hydrogen bonds are predicted according to the interatomic distance, bond angle and atom types. Hydrogen bonds are predicted if a hydrogen is in the range from 1.2 to 2.76 Å of a “compatible” donor atom.

## 5. Conclusions

In conclusion, we found eight polymorphisms of the canine *PRNP* gene, including six novel polymorphisms and identified strong LDs and six major haplotypes among eight polymorphisms. Additionally, we compared the distribution of most haplotype 1 of the canine *PRNP* gene among eight dog breeds. The distribution of haplotypes was significantly different among the eight dog breeds. In addition, the number of identified polymorphisms was different in each dog breed. Furthermore, we estimated the biological impact of nonsynonymous SNPs and insertion/deletion using *in silico* 4 programs. The polymorphisms, including 64_71delHGGGWGQP, Asp182Glu, and Asp182Gly, were predicted to be deleterious, and two polymorphisms, including Asp163Glu and Asp182Gly, had more aggregation propensity than wild-type dog PrP. Finally, we predicted the 3D structure and hydrogen bonds of dog PrP according to alleles of nonsynonymous SNPs. The number of hydrogen bonds of dog PrP with alleles Glu182 and Gly182 were predicted to be three less than that of dog PrP with allele Asp182. Based on these results, we suggest that nonsynonymous SNPs, including Asp182Glu and Asp182Gly can affect the stability of dog PrP.

## Figures and Tables

**Table 1:** Genotype and allele frequencies of the *PRNP* polymorphisms in dogs.

Polymorphisms		Genotype Frequency, *n* (%)			Allele frequency, *n* (%)		HWE
c.190_213del	64_71del HGGGWGQP	Wt/Wt 201 (98.5)	Wt/Del 3 (1.5)	Del/Del 0 (0)	Wt 405 (99.3)	Del 3 (0.7)	1.0
c.198T>C	Gly66Gly	T/T 159 (77.9)	T/C 33 (16.2)	C/C 12 (5.9)	T 351 (86.0)	C 57 (14.0)	<0.001
c.301A>G	Ser101Gly	A/A 112 (54.9)	A/G 73 (35.8)	G/G 19 (9.3)	A 297 (72.8)	G 111 (27.2)	0.2168
c.372G>A	Ala124Ala	G/G 196 (96.1)	G/A 8 (3.9)	A/A 0 (0)	G 400 (98.0)	A 8 (2.0)	1.0
c.489C>G	Asp163Glu	C/C 125 (61.3)	C/G 62 (30.4)	G/G 17 (8.3)	C 312 (76.5)	G 96 (23.5)	0.0432
c.545A>G	Asp182Gly	A/A 202 (99.0)	A/G 2 (1.0)	G/G 0 (0)	A 406 (99.5)	G 2 (0.5)	1.0
c.546C>A	Asp182Glu	C/C 200 (98.0)	C/A 2 (1.0)	A/A 2 (1.0)	C 402 (98.5)	A 6 (1.5)	<0.001
c.729T>C	Pro243Pro	T/T 120 (58.8)	T/C 63 (30.9)	C/C 21 (10.3)	T 303 (74.3)	C 105 (25.7)	0.011

**Table 2:** Haplotype frequencies of 8 *PRNP* polymorphisms in the canine *PRNP* gene.

Haplotypes	c.190_213del	c.198T>C	c.301A>G	c.372G>A	c.489C>G	c.545A>G	c.546C>A	c.729T>C	Frequency (*n* = 204)
ht1	Wt	T	A	G	C	A	C	T	284 (0.696)
ht2	Wt	T	G	G	G	A	C	C	56 (0.137)
ht3	Wt	C	G	G	G	A	C	C	36 (0.088)
ht4	Wt	C	G	G	C	A	C	T	7 (0.017)
ht5	Wt	C	G	A	C	A	C	C	6 (0.015)
ht6	Wt	T	A	G	C	A	A	T	5 (0.012)
others ^a^									14 (0.035)

^a^ Others contain rare haplotypes with frequency <0.012.

**Table 3:** Linkage disequilibrium (LD) of 8 *PRNP* polymorphisms in dogs.

	∣D’∣							
*r^2^*	c.190_213del	c.198T>C	c.301A>G	c.372G>A	c.489C>G	c.545A>G	c.546C>A	c.729T>C
c.190_213del	-	1.0	1.0	1.0	1.0	1.0	1.0	1.0
c.198T>C	0.046	-	0.919	1.0	0.55	0.308	1.0	0.688
c.301A>G	0.002	0.367	-	1.0	0.985	0.263	0.387	0.96
c.372G>A	0	0.123	0.054	-	1.0	1.0	1.0	0.574
c.489C>G	0.024	0.16	0.799	0.006	-	0.056	0.292	1.0
c.545A>G	0	0.003	0.0	0	0	-	1.0	0.089
c.546C>A	0	0.002	0.001	0	0.0	0.0	-	0.352
c.729T>C	0.021	0.222	0.855	0.019	0.888	0.0	0.001	-

**Table 4:** Different distributions of *PRNP* polymorphisms in 8 dog breeds.

Breeds (n)	Polymorphisms						Total Number		
Maltese (77)	64_71delHGGGWGQP	Gly66Gly	Ser101Gly	Ala124Ala	Asp163Glu	Asp182Gly	Asp182Glu	Pro243Pro	8
Shih Tzu (29)		Gly66Gly	Ser101Gly	Ala124Ala	Asp163Glu			Pro243Pro	5
Toy Poodle (25)	64_71delHGGGWGQP	Gly66Gly	Ser101Gly	Ala 124Ala	Asp163Glu			Pro243Pro	6
Yorkshire Terrier (19)							Asp182Glu		1
Pomeranian (15)		Gly66Gly	Ser101Gly		Asp163Glu			Pro243Pro	4
Chihuahua (11)		Gly66Gly	Ser101Gly		Asp163Glu	Asp182Gly	Asp182Glu	Pro243Pro	6
Schnauzer (7)		Gly66Gly	Ser101Gly		Asp163Glu			Pro243Pro	4
Bichon Frise (5)			Ser101Gly		Asp163Glu			Pro243Pro	3
Mixed dog (16)		Gly66Gly	Ser101Gly	Ala 124Ala	Asp163Glu		Asp182Glu	Pro243Pro	6

**Table 5:** *In silico* analysis of nonsynonymous SNPs of the *PRNP* gene in dogs.

Polymorphisms		PolyPhen-2		PROVEAN		PANTHER	
		Score	Prediction	Score	Prediction	Score	Prediction
c.190_213del	*p*.64_71del HGGGWGQP		* NA	−13.135	Deleterious		* NA
c.301A>G	Ser101Gly	0.000	Benign	−0.260	Neutral	85	Probably benign
c.489C>G	Asp163Glu	0.001	Benign	−0.194	Neutral	85	Probably benign
c.545A>G	Asp182Glu	0.999	Probably damaging	−1.452	Neutral	361	Possibly damaging
c.546C>A	Asp182Gly	1	Probably damaging	−1.909	Neutral	361	Possibly damaging

* NA, Not available.

## Abbreviations

PRNP	Prion protein gene
TSEs	Transmissible spongiform encephalopathies
LD	Linkage disequilibrium
PrP	Prion protein
SNPs	Single nucleotide polymorphisms
BSE	Bovine spongiform encephalopathy
MDCK	Madin–Darby canine kidney
PMCA	Protein misfolding cyclic amplification
CWD	Chronic wasting disease
RML	Rocky Mountain Laboratory
CJD	Creutzfeldt–Jakob disease
ORF	Open reading frame
HWE	Hardy-Weinberg equilibrium
IC	Intracerebral
IP	Intraperitoneal
NZW	New Zealand White
EtBr	Ethidium bromide
FPR	False positive rate
NMR	Nuclear magnetic resonance
